# Retracted: Effect of Respiration Training-Assisted Western Medicine Therapy on Activity Tolerance, Pulmonary Function, and Quality of Life of Chronic Obstructive Pulmonary Disease Patients in the Stable Phase

**DOI:** 10.1155/2023/9865734

**Published:** 2023-01-26

**Authors:** Journal of Healthcare Engineering

*Journal of Healthcare Engineering* has retracted the article titled “Effect of Respiration Training-Assisted Western Medicine Therapy on Activity Tolerance, Pulmonary Function, and Quality of Life of Chronic Obstructive Pulmonary Disease Patients in the Stable Phase” [1] due to concerns that the peer review process has been compromised.

Following an investigation conducted by the Hindawi Research Integrity team [2], significant concerns were identified with the peer reviewers assigned to this article; the investigation has concluded that the peer review process was compromised. We therefore can no longer trust the peer review process, and the article is being retracted with the agreement of the Chief Editor.

## References

[1] Tong Y., Cui J., Chai D. (2022). Effect of Respiration Training-Assisted Western Medicine Therapy on Activity Tolerance, Pulmonary Function, and Quality of Life of Chronic Obstructive Pulmonary Disease Patients in the Stable Phase. *Journal of Healthcare Engineering*.

[2] Ferguson L. (2022). Advancing Research Integrity Collaboratively and with Vigour. https://www.hindawi.com/post/advancing-research-integrity-collaboratively-and-vigour/.

